# Hybrid Shell-Beam Inverse Finite Element Method for the Shape Sensing of Stiffened Thin-Walled Structures: Formulation and Experimental Validation on a Composite Wing-Shaped Panel

**DOI:** 10.3390/s23135962

**Published:** 2023-06-27

**Authors:** Marco Esposito, Rinto Roy, Cecilia Surace, Marco Gherlone

**Affiliations:** 1 Department of Mechanical and Aerospace Engineering, Politecnico di Torino, 10129 Torino, Italy; marco.esposito@polito.it; 2 Department of Structural, Geotechnical and Building Engineering, Politecnico di Torino, 10129 Torino, Italy; rinto.roy@polito.it (R.R.); cecilia.surace@polito.it (C.S.)

**Keywords:** shape sensing, mindlin plate, timoshenko beam, carbon-epoxy panel, fiber optics, structural health monitoring, inverse problem

## Abstract

This work presents a novel methodology for the accurate and efficient elastic deformation reconstruction of thin-walled and stiffened structures from discrete strains. It builds on the inverse finite element method (iFEM), a variationally-based shape-sensing approach that reconstructs structural displacements by matching a set of analytical and experimental strains in a least-squares sense. As iFEM employs the finite element framework to discretize the structural domain and as the displacements and strains are approximated using element shape functions, the kind of element used influences the accuracy and efficiency of the iFEM analysis. This problem is addressed in the present work through a novel discretization scheme that combines beam and shell inverse elements to develop an iFEM model of the structure. Such a hybrid discretization paradigm paves the way for more accurate shape-sensing of geometrically complex structures using fewer sensor measurements and lower computational effort than traditional approaches. The hybrid iFEM is experimentally demonstrated in this work for the shape sensing of bending and torsional deformations of a composite stiffened wing panel instrumented with strain rosettes and fiber-optic sensors. The experimental results are accurate, robust, and computationally efficient, demonstrating the potential of this hybrid scheme for developing an efficient digital twin for online structural monitoring and control.

## 1. Introduction

Inverse problems are concerned with estimating unknown characteristics or parameters of a structural system from indirect observations. Such problems are often ill-posed, implying that conditions of the existence and uniqueness of a solution are not satisfied. Additionally, small errors in the measured data can lead to a large error in the estimated model parameters, highlighting the instability of the solution achieved. Nevertheless, inverse problems are of great importance in structural engineering, with structural health monitoring (SHM) [1] perhaps the most obvious and direct application. In the case of SHM, the inverse problem can be formulated as the estimation of the health state of a structure using real-time strain, acceleration, or temperature measurements from a discrete set of sensors instrumented on the structure [2]. Such a real-time monitoring paradigm can aid in efficiently planning maintenance activities, reducing operational cost and human effort, and improving overall structural safety [3]. The inverse problem of shape sensing, i.e., real-time reconstruction of structural displacements from discrete strains, is highly relevant in this regard [4]. The reconstructed displacements, strains, or stresses can be used for damage diagnosis or prognosis, acting as an efficient digital twin for real-time structural monitoring applications. Additionally, shape-sensing techniques can be instrumental in developing efficient monitoring and control approaches for future shape morphing structures [5].

Solution approaches for the inverse shape-sensing problem have been the focus of numerous works [6], with the major efforts briefly discussed and compared here. Structural displacements and rotations can be reconstructed by integrating experimental strains measured by a discrete set of sensors [7]. This approach, founded on the classical beam or plate theories, has been developed for one-dimensional (1D) beam [8,9] and two-dimensional (2D) plate structures [10] and applied to aeroelastic shape control applications [11]. Alternatively, the structural displacement field can be modeled as a weighted superposition of basis functions [12,13,14]. This approach requires an a priori selection of basis functions, such as piecewise polynomials [15] or the vibrational mode shapes of the structure [16,17,18], and the displacement field is obtained by using strain measurements to compute the corresponding weights. Depending on the availability of a large displacement and strain measurement database, the use of neural networks [19] also offers a purely data-driven solution to this inverse problem. Finally, shape-sensing approaches based on a variational principle round up this discussion, the major work being the inverse finite element method (iFEM) proposed by Tessler and Spangler [20,21]. Among these approaches, iFEM has perhaps received the widest attention and acceptance, as it requires fewer strain measurements than integration-based approaches, is independent of basis function selection or prior knowledge of structural modal properties, and requires no computationally intensive model training to produce reliable predictions [6].

Shape sensing using the iFEM is based on the finite element discretization framework, where the structural domain is discretized using finite elements. The element displacement field is approximated by using an interpolation based on shape functions and nodal degrees-of-freedom (DOF), while the strains are obtained using the linear strain-displacement relations. Displacement reconstruction is based on solving an error functional described as the least-squares error between analytically and experimentally evaluated strains [20,21]. As it is based on the linear strain-displacement relations, the iFEM is inherently independent of the structure’s material properties or operational conditions, presenting a model-independent approach for real-time deformation monitoring. Based on the structural geometry investigated and the kinematic assumptions used, existing iFEM developments can broadly be classified into 1D and 2D formulations.

Preliminary iFEM development focused on the shape sensing of 2D plate or shell structures [22]. The inverse shell elements developed include the three-node shell, iMIN3 [22], four-node shell, iQS4 [23] (featuring a drilling DOF), and the eight-node curved shell, iCS8 [24], elements. These elements are based on Mindlin theory [25], with the former two using C${}^{0}$-continuous anisoparametric interpolations [26], producing an improved treatment of transverse shear in the thin plate regime. These efforts also extend to elements for analyzing multi-layered composite or sandwich structures [27]. This inverse element repository has been successfully applied for various numerical and experimental shape-sensing studies featuring metallic and composite structures. The structures investigated include wing-shaped geometries [6,28,29], stiffened panels [30,31,32], wing boxes [33], and marine structures [34,35]. Moreover, the application of iFEM has been extended to geometrically non-linear problems in [36,37]. The success of these investigations has also prompted the use of iFEM for SHM applications, specifically for damage detection in metallic [38,39] and composite [40,41,42] structures, and more recently for damage prognosis [43,44]. A key limitation of the 2D iFEM is the need for a large number of strain measurements to generate accurate results, which can be resolved using an optimal selection of sensor locations [45,46,47] and strain pre-extrapolation to produce virtual measurement sites [48,49,50,51].

The inefficiency of the 2D iFEM to accurately analyze beam and frame structures inspired the development of the 1D iFEM by Gherlone et al. [52]. The preliminary formulation is based on Timoshenko beam kinematics. The displacement field is obtained by solving a least-squares error functional defined between the analytical and experimental sectional strains, representing the beam’s axial, bending, transverse shear, and torsional deformation. Later works feature formulations for slender beams [53], handling cross-sectional complexities [54,55], and composite structures [56]. Numerical and experimental application of the 1D iFEM feature the monitoring of circular and airfoil beams [54,57], radio telescope reflectors [58], wing structures [59], subsea pipelines [60], etc. Recent efforts have also been aimed at non-linear deformation monitoring [61] and optimal sensor placement [46,47,62] for efficient shape sensing.

At this stage, it is useful to consider the advantages and limitations of both the 1D and 2D iFEM approaches discussed in this section. The 1D iFEM, using a low-fidelity finite element discretization of inverse beam elements, can reconstruct structural displacements using a relatively low number of strain sensors with low computational effort. However, the results obtained are also of a low resolution or accuracy, i.e., the nodal displacements are reconstructed along the beam axis, and 3D displacement reconstruction depends on the accuracy of the kinematic assumptions. These assumptions can break down in the case of complex structures, such as a wing box, leading to inaccurate results. In contrast, the high-fidelity inverse shell element discretization employed by the 2D iFEM allows for more accurate and precise structural modeling. However, the analysis is computationally intensive, requiring relatively more strain-sensor measurements to produce accurate results. This work combines the 1D and 2D approaches using a hybrid scheme that discretizes the structure using beam and shell finite elements. Such a coupling enlarges the repository of inverse elements available to model a structure, generating greater accuracy than the 1D iFEM but at a lower computational cost and requiring fewer sensors than the 2D iFEM. The novel hybrid iFEM is demonstrated in this work for the experimental shape sensing of a composite stiffened wing panel instrumented with strain rosettes and fiber-optic sensors. The inverse model of the stiffened panel is developed using beam elements to model the stringers and shell elements to model the flat panel or skin. The merits and limitations of the hybrid iFEM are qualified by comparing shape-sensing results to those obtained from a complete shell model. Moreover, this work also assesses the robustness of the iFEM in using a constant strain-sensor configuration to produce accurate results across a variety of panel deformations. A sensor configuration, optimized for a specific load configuration, is selected for this purpose, and experimental results are computed for different panel load cases to test the adaptability of the approach.

The paper is organized as follows: Section 2 briefly recounts the theoretical formulation of the 1D and 2D iFEM and the development of the hybrid iFEM by integrating these two approaches. Section 3 presents the composite stiffened panel used for the experimental tests, along with details of the inverse models, sensor configurations, and the load cases used. Results of the experimental validation efforts of the hybrid iFEM are presented in Section 4. Finally, Section 5 ends with the main conclusions and opportunities for future work.

## 2. Hybrid Inverse Finite Element Method

The hybrid approach introduced in this work is based on the 1D and 2D iFEM formulations of Gherlone et al. [52] and Tessler et al. [21]. These two iFEM approaches are initially discussed in this section. Subsequently, the theoretical formulation of the hybrid iFEM is presented.

### 2.1. 1D Inverse Finite Element Method

**Figure 1:**
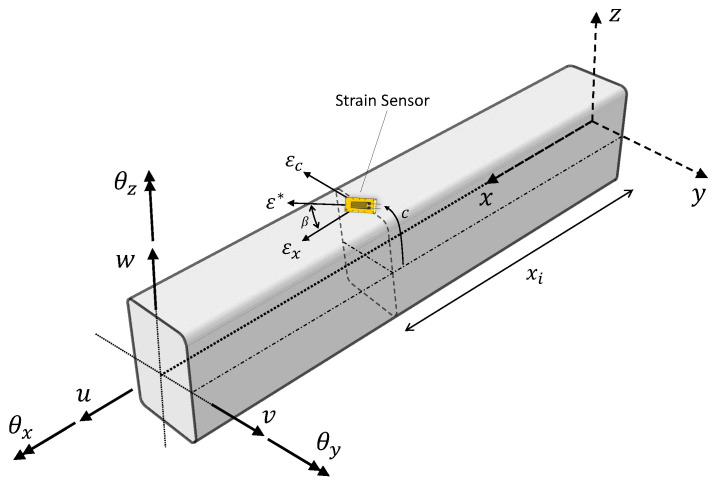
Definition of the variables used to describe Timoshenko beam kinematics; the location and component of strains measured by a sensor placed on the beam surface are also shown.

Consider a prismatic beam defined in the 3D Cartesian coordinates, x≡(x,y,z), with the coordinate origin situated at the beam root (as shown in Figure 1). The *x*-axis is parallel to the beam axis and coincident with the shear center of the section, while *y* and *z* are the two transverse axes. The beam has a length *L*, cross-sectional area $A_{b}$, second area moments $I_{yy}$ and $I_{zz}$, torsional constant $I_{t}$, and is made of a homogeneous isotropic material with Young’s modulus *E*, shear modulus *G*, and Poisson’s ratio ν.

#### 2.1.1. Kinematic Relations

The 1D iFEM for beam or frame structures is based on the kinematic assumptions of Timoshenko beam theory [52]. The Cartesian components of the displacement vector are described in terms of the kinematic variables $u_{b}\equiv {\left\{u,v,w,\theta_{x},\theta_{y},\theta_{z}\right\}}^{T}$ as

(1) $$\begin{array}{c} u_{x}\left(x\right)=u\left(x\right)+z\theta_{y}\left(x\right)-y\theta_{z}\left(x\right),\;u_{y}\left(x\right)=v\left(x\right)-z\theta_{x}\left(x\right),\;u_{z}\left(x\right)=w\left(x\right)+y\theta_{x}\left(x\right) \end{array}$$

where $u_{x}$, $u_{y}$, and $u_{z}$ are the displacements of any material point along the *x*, *y*, and *z*-axes, respectively. The variables *u*, *v*, and *w* are the beam displacements at the shear center along the *x*, *y*, and *z*-axes, respectively (see Figure 1), while $\theta_{x}$, $\theta_{y}$, and $\theta_{z}$ are the torsional and bending rotations about the *x*, *y*, and *z*-axes, respectively.

Using the linear strain-displacement relations, the strain field is computed from the kinematic field of Equation (1), and is given as

(2) $$\left\{\begin{array}{c} \varepsilon_{x}\left(x\right)\\ \gamma_{xz}\left(x\right)\\ \gamma_{xy}\left(x\right) \end{array}\right\}=\left\{\begin{array}{c} u_{x,x}\\ u_{x,z}+u_{z,x}\\ u_{x,y}+u_{y,x} \end{array}\right\}=\left\{\begin{array}{c} e_{1}\left(x\right)+ze_{2}\left(x\right)+ye_{3}\left(x\right)\\ e_{4}\left(x\right)+ye_{6}\left(x\right)\\ e_{5}\left(x\right)-ze_{6}\left(x\right) \end{array}\right\}$$

where $e_{i}$ (i=1,…,6) are the sectional strains defined along the beam axis and represent the axial stretching, bending curvatures, transverse shear, and torsional strain of a beam section. The displacements and strains within an inverse element are approximated by interpolating the nodal DOF using element shape functions. The sectional strains so computed (’analytically’) can be written in vector form as

(3) $$\begin{array}{cc} e(u_{b}) & ={\left\{e_{1},e_{2},e_{3},e_{4},e_{5},e_{6}\right\}}^{T}\\ \; & ={\left\{u_{,x},\theta_{y,x},-\theta_{z,x},w_{,x}+\theta_{y},v_{,x}-\theta_{z},\theta_{x,x}\right\}}^{T}=B^{s}u_{b}^{e} \end{array}$$

where $B^{s}$ is a matrix of shape function derivatives, and $u_{b}^{e}$ is the vector of nodal DOF of element *e* of the inverse model.

#### 2.1.2. Experimental Sectional Strains

The sectional strains of Equation (3) can be computed experimentally using strain measurements from sensors mounted on the beam surface. For a beam instrumented with a discrete set of strain gauges or fiber-optic sensors, the sensor locations are defined by $x_{i}$ (i=1,…,N), while β defines sensor orientation with respect to the beam axis (see Figure 1). For any general beam profile, the surface strain measurement $\varepsilon^{*}$, described in terms of the experimental sectional strains $e_{j}^{\epsilon }$ (j=1−6), is given by [54]

(4) $$\begin{array}{cc} \varepsilon^{*}\left(x_{i},c,\beta \right) & =\left(e_{1}^{\epsilon }\left(x_{i}\right)+e_{2}^{\epsilon }\left(x_{i}\right)z\left(c\right)+e_{3}^{\epsilon }\left(x_{i}\right)y\left(c\right)\right)\left(\cos^{2}\beta -\nu \sin^{2}\beta \right)+\\ \; & \left(\frac{1}{k_{\epsilon z}}e_{4}^{\epsilon }\left(x_{i}\right)f_{1}\left(c\right)+\frac{1}{k_{\epsilon y}}e_{5}^{\epsilon }\left(x_{i}\right)f_{2}\left(c\right)+e_{6}^{\epsilon }\left(x_{i}\right)f_{3}\left(c\right)\right)\cos\beta \sin\beta \end{array}$$

where *c* is the circumferential coordinate, $f_{1}$, $f_{2}$, and $f_{3}$ are functions that describe the surface tangential shear strain variation due to transverse and torsional loads, and $k_{\epsilon y}$ and $k_{\epsilon z}$ are shear coefficients defined by the beam profile. A detailed derivation of Equation (4) and the computation of shear coefficients and functions for a beam profile are given in Ref. [54].

#### 2.1.3. Least-Squares Error Functional

The 1D iFEM is based on the finite element discretization framework, where the structural domain is discretized using beam finite elements with element lengths, $l_{e}$. For each inverse element, an error functional can be written as

(5) $$\begin{array}{c} \Phi_{b}^{e}\left(u_{b}^{e}\right)\equiv w_{s}\Phi_{s}\left(u_{b}^{e}\right) \end{array}$$

where $w_{s}$ is a vector of weighing coefficients. The vector of functionals $\Phi_{s}=\left\{\Phi_{k}^{e}\right\}$ (k=1,...,6) captures the least-squares error between the analytical and experimental sectional strains computed at *N* axial sections and is given by

(6) $$\Phi_{k}^{e}=\frac{l_{e}}{N}\sum_{i=1}^{N}{\left[e_{k}\left(x_{i}\right)-{\left(e_{k}^{\epsilon }\right)}_{i}\right]}^{2}$$

The weighing coefficients control the enforcement of the least-squares compatibility of each sectional strain component and ensure the dimensional consistency of Equation (5). For an inverse beam element formulated based on Timoshenko beam theory, the weighting coefficient vector $w_{s}=\left\{w_{k}\right\}$ (k=1,…,6) is written as

(7) $$w_{s}=\left\{w_{k}\right\}=\left\{w_{1}^{o},w_{2}^{o}\left(I_{yy}/A_{b}\right),w_{3}^{o}\left(I_{zz}/A_{b}\right),w_{4}^{o},w_{5}^{o},w_{6}^{o}\left(I_{t}/A_{b}\right)\right\}$$

where $w_{k}^{0}$ (k=1,…,6) are the dimensionless weighting coefficients.

The element error functional is solved by minimizing Equation (5) with respect to the nodal DOF of the element to obtain the following set of linear algebraic equations

(8) $$\frac{\partial \Phi_{b}^{e}\left(u_{b}^{e}\right)}{\partial u_{b}^{e}}=k_{b}^{e}u_{b}^{e}-f_{b}^{e}=0\to k_{b}^{e}u_{b}^{e}=f_{b}^{e}$$

where matrix $k_{b}^{e}$ and vector $f_{b}^{e}$ are analogous to the element stiffness matrix and force vector in the direct FEM. Here, $k_{b}^{e}$ is only a function of the sensor positions, while $f_{b}^{e}$ is a function of the sensor positions and measured strains. These element matrices are the weighted sum of contributions from each sectional strain term and can be written as

(9) $$\begin{array}{c} k_{b}^{e}\left(u_{b}^{e}\right)=\sum_{k=1}^{6}w_{k}\left(\frac{l_{e}}{N}\sum_{i=1}^{N}{\left(B_{k}^{s}\left(x_{i}\right)\right)}^{T}B_{k}^{s}\left(x_{i}\right)\right),\;f_{b}^{e}\left(u_{b}^{e}\right)=\sum_{k=1}^{6}w_{k}\left(\frac{l_{e}}{N}\sum_{i=1}^{N}{\left(B_{k}^{s}\left(x_{i}\right)\right)}^{T}{\left(e_{k}^{\epsilon }\right)}_{i}\right) \end{array}$$

### 2.2. 2D Inverse Finite Element Method

**Figure 2:**
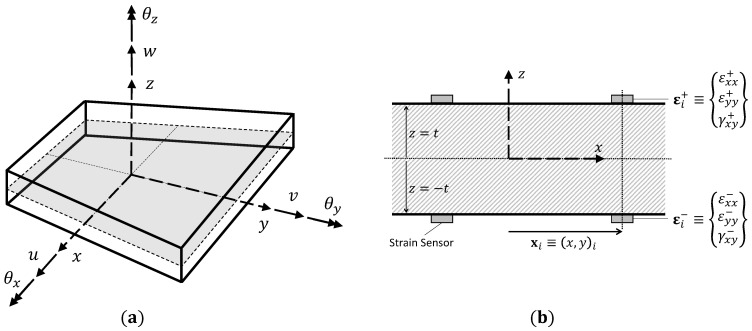
Illustration of the plate structure: (**a**) kinematic variables used to describe the plate deformations, and (**b**) sensors mounted on the top and bottom plate surfaces.

Consider a plate or shell structure defined in the 3D Cartesian coordinate frame $\left(x,y,z\right)\subset R^{3}$ (shown in Figure 2). The orthogonal coordinates, x≡(x,y), define the plate mid-plane with the *z*-axis along the plane normal (z=0 defines the mid-plane surface). The plate has a thickness 2t, where z∈[−t,t] and mid-plane area $A_{p}$.

#### 2.2.1. Kinematic Relations

The 2D iFEM for plates or shells is formulated based on the kinematic assumptions of Mindlin theory [25]. The Cartesian components of the displacement vector can be described in terms of the kinematic variables $u_{p}\equiv {\left\{u,v,w,\theta_{x},\theta_{y}\right\}}^{T}$ as

(10) $$u_{x}=u\left(x\right)+z\theta_{y}\left(x\right),\;u_{y}=v\left(x\right)-z\theta_{x}\left(x\right),\;u_{z}=w\left(x\right)$$

where the kinematic variables *u* and *v* are the mid-plane surface displacements in the *x* and *y* directions; *w* is the transverse deflection averaged across the plate thickness; and $\theta_{x}$ and $\theta_{y}$ are the section rotations about the *x* and *y*-axes, respectively (see Figure 2).

Using the linear strain-displacement relation, the strain field of the plate is computed from Equation (10) and has the following form

(11) $$\left\{\begin{array}{c} \varepsilon_{xx}\\ \varepsilon_{yy}\\ \gamma_{xy} \end{array}\right\}=\left\{\begin{array}{c} u_{x,x}\\ u_{y,y}\\ u_{x,y}+u_{y,x} \end{array}\right\}=\left\{\begin{array}{c} u_{,x}\\ v_{,y}\\ u_{,y}+v_{,x} \end{array}\right\}+z\left\{\begin{array}{c} \theta_{y,x}\\ -\theta_{x,y}\\ -\theta_{x,x}+\theta_{y,y} \end{array}\right\}=m\left(u_{p}\right)+zk\left(u_{p}\right)$$

where m and k are the strain measures defined in the plate mid-plane surface, representing the in-plane stretching and curvature of the mid-plane. Similarly, the transverse shear strains of the plate given by Mindlin theory are written as

(12) $$\left\{\begin{array}{c} \gamma_{xz}\\ \gamma_{yz} \end{array}\right\}=\left\{\begin{array}{c} u_{z,x}+u_{x,z}\\ u_{z,y}+u_{y,z} \end{array}\right\}=\left\{\begin{array}{c} w_{,x}+\theta_{y}\\ w_{,y}-\theta_{x} \end{array}\right\}=g\left(u_{p}\right)$$

where g represents the transverse shear strain measures of the plate.

Similar to the 1D iFEM, the displacements and strains within an element are approximated using element shape functions to obtain the following relations

(13) $$\left\{\begin{array}{c} \varepsilon_{xx}\\ \varepsilon_{yy}\\ \gamma_{xy} \end{array}\right\}=m\left(u_{p}^{e}\right)+zk\left(u_{p}^{e}\right)=B^{m}u_{p}^{e}+zB^{k}u_{p}^{e},\;\left\{\begin{array}{c} \gamma_{xz}\\ \gamma_{yz} \end{array}\right\}=g\left(u_{p}^{e}\right)=B^{g}u_{p}^{e}$$

where $B^{m}$, $B^{k}$, and $B^{g}$ are matrices of shape function derivatives corresponding to the membrane, curvature, and transverse shear strain measures, respectively, and $u_{p}^{e}$ is the vector of nodal DOF of the element. Although the kinematic variables of Mindlin theory do not enlist $\theta_{z}$, it is taken into account as one of the DOF of the inverse element (see Figure 2).

#### 2.2.2. Experimental Strain Measures

The strain measures of Equation (13) can be computed experimentally using strain measurements from sensors mounted on the top (z=t) and bottom (z=−t) surfaces of the plate. Assuming a plate instrumented with a discrete number of strain gauges or fiber sensors, the sensor locations are defined by $x_{i}={\left(x,y\right)}_{i}$, where i=1,…,N (see Figure 2), while the strains measured on the top and bottom surfaces are $\varepsilon_{i}^{+}={\left\{\varepsilon_{xx}^{+},\varepsilon_{yy}^{+},\gamma_{xy}^{+}\right\}}_{i}^{T}$ and $\varepsilon_{i}^{-}={\left\{\varepsilon_{xx}^{-},\varepsilon_{yy}^{-},\gamma_{xy}^{-}\right\}}_{i}^{T}$, respectively. The surface strain measurements are used to compute the strain measures on the plate’s mid-plane surface using the relations

(14) $$m_{i}^{\epsilon }=\frac{1}{2}{\left(\left\{\begin{array}{c} \epsilon_{xx}^{+}\\ \epsilon_{yy}^{+}\\ \gamma_{xy}^{+} \end{array}\right\}+\left\{\begin{array}{c} \epsilon_{xx}^{-}\\ \epsilon_{yy}^{-}\\ \gamma_{xy}^{-} \end{array}\right\}\right)}_{i},\;k_{i}^{\epsilon }=\frac{1}{2t}{\left(\left\{\begin{array}{c} \epsilon_{xx}^{+}\\ \epsilon_{yy}^{+}\\ \gamma_{xy}^{+} \end{array}\right\}-\left\{\begin{array}{c} \epsilon_{xx}^{-}\\ \epsilon_{yy}^{-}\\ \gamma_{xy}^{-} \end{array}\right\}\right)}_{i}$$

The transverse shear strain measures cannot be computed directly from experimental strains and are omitted in this work. The procedure used to handle such situations is discussed in the following section.

#### 2.2.3. Least-Squares Error Functional

The 2D iFEM is based on discretizing the structural domain using inverse finite elements with elemental areas $A_{p}^{e}$. For each inverse element *e*, a weighted least-squares error functional between the analytical and experimental strain measures is defined as

(15) $$\begin{array}{c} \Phi_{p}^{e}\left(u_{p}^{e}\right)\equiv w_{m}\Phi_{m}\left(u_{p}^{e}\right)+w_{k}\Phi_{k}\left(u_{p}^{e}\right)+w_{g}\Phi_{g}\left(u_{p}^{e}\right) \end{array}$$

where $w_{m}$, $w_{k}$, and $w_{s}$, are row vectors of weighting coefficients used to enforce the correlation between the analytical and experimental strain measures in the error functional. The individual error functionals, $\Phi_{m}$, $\Phi_{k}$, and $\Phi_{s}$ correspond to the membrane, curvature, and transverse shear strain measures, respectively, and are given as

(16) $$\begin{array}{cc} \Phi_{m} & \equiv \frac{1}{A_{p}^{e}}\int_{A_{p}^{e}}{\left[m\left(u_{p}^{e}\right)-m^{\epsilon }\right]}^{2}dA,\;\Phi_{k}\equiv \frac{{\left(2t\right)}^{2}}{A_{p}^{e}}\int_{A_{p}^{e}}{\left[k\left(u_{p}^{e}\right)-k^{\epsilon }\right]}^{2}dA\\ \Phi_{g} & \equiv \frac{1}{A_{p}^{e}}\int_{A_{p}^{e}}{\left[g\left(u_{p}^{e}\right)-g^{\epsilon }\right]}^{2}dA \end{array}$$

If experimental strains are available for an element, the corresponding weighting coefficients are set to unity ($w_{m}=w_{k}=\left\{1,1,1\right\}$ and $w_{g}=\left\{1,1\right\}$), while in the absence of measurements, they are set to a small value ($10^{-5}-10^{-3}$). The use of a lower weight reduces the element contribution to the global error functional. As $g^{\epsilon }$ cannot be computed directly from experimental measurements, the following squared norm form is used

(17) $$\begin{array}{c} \Phi_{g}\equiv \frac{1}{A_{p}^{e}}\int_{A_{p}^{e}}{\left[g\left(u_{p}^{e}\right)\right]}^{2}dA \end{array}$$

where the corresponding weighting coefficient vector is set to a small value, $w_{g}=\left\{10^{-5},10^{-5}\right\}$.

The element error function is solved by minimizing Equation (15) with respect to the nodal DOF of the element to obtain the following set of linear algebraic equations

(18) $$\frac{\partial \Phi_{p}^{e}\left(u_{p}^{e}\right)}{\partial u_{p}^{e}}=k_{p}^{e}u_{p}^{e}-f_{p}^{e}=0\to k_{p}^{e}u_{p}^{e}=f_{p}^{e}$$

where matrix $k_{p}^{e}$ and vector $f_{p}^{e}$ are given in terms of the shape function derivatives as

(19) $$\begin{array}{cc} k_{p}^{e}\left(u_{p}^{e}\right) & =\frac{1}{A_{p}^{e}}\int_{A_{p}^{e}}\left[w_{m}{\left(B^{m}\right)}^{T}B^{m}+w_{k}{\left(2t\right)}^{2}{\left(B^{k}\right)}^{T}B^{k}+w_{g}{\left(B^{g}\right)}^{T}B^{g}\right]dA,\\ f_{p}^{e}\left(u_{p}^{e}\right) & =\frac{1}{A_{p}^{e}}\int_{A_{p}^{e}}\left[w_{m}{\left(B^{m}\right)}^{T}m^{\epsilon }+w_{k}{\left(2t\right)}^{2}{\left(B^{k}\right)}^{T}k^{\epsilon }+w_{g}{\left(B^{g}\right)}^{T}g^{\epsilon }\right]dA. \end{array}$$

### 2.3. Hybrid Formulation

The hybrid iFEM formulation combines both 1D and 2D approaches by discretizing the structure using a combination of beam and shell inverse finite elements. For example, in the case of a stiffened panel, the stringers are modeled using beam elements and the panel or skin using shell elements. For each beam or shell element, the local element matrices can be converted into the global coordinate system using a transformation defined as

(20) $$\begin{array}{cc} {\left\{k_{b}^{e}\right\}}_{g} & ={\left(T^{e}\right)}^{T}k_{b}^{e}T^{e},\;{\left\{f_{b}^{e}\right\}}_{g}={\left(T^{e}\right)}^{T}f_{b}^{e}\\ {\left\{k_{p}^{e}\right\}}_{g} & ={\left(T^{e}\right)}^{T}k_{p}^{e}T^{e},\;{\left\{f_{p}^{e}\right\}}_{g}={\left(T^{e}\right)}^{T}f_{p}^{e} \end{array}$$

where the transformation matrix, $T^{e}$, takes into account the local element orientation and offsets with respect to the global coordinate system.

The transformation contribution due to the offsets is computed based on the kinematic relations of first-order shear deformation theory (see Equations (1) and (10)). For any node of an inverse beam or plate element, the local DOF can be related to the global DOF using the relations

(21) $${\left\{\begin{array}{c} u\\ v\\ w\\ \theta_{x}\\ \theta_{y}\\ \theta_{z} \end{array}\right\}}_{l}=\left[\begin{array}{cccccc} 1 & 0 & 0 & 0 & z_{0} & -y_{0}\\ 0 & 1 & 0 & -z_{0} & 0 & 0\\ 0 & 0 & 1 & y_{0} & 0 & 0\\ 0 & 0 & 0 & 1 & 0 & 0\\ 0 & 0 & 0 & 0 & 1 & 0\\ 0 & 0 & 0 & 0 & 0 & 1 \end{array}\right]{\left\{\begin{array}{c} u\\ v\\ w\\ \theta_{x}\\ \theta_{y}\\ \theta_{z} \end{array}\right\}}_{g}$$

where $y_{0}$ and $z_{0}$ define the offset of the local beam element axis with respect to the global frame. Similarly, for shell elements, $z_{0}$ is the offset of the mid-plane surface with respect to the global frame (with $y_{0}=0$).

Using the standard finite element assembly procedure, the contributions from all beam and shell elements are assembled to obtain the global set of equations of the structure

(22) KU=F

As in the direct FEM, nodal boundary conditions are applied to constrain the structure against rigid-body motion and ensure a non-singular system matrix. In the case of overlapping nodes, the associated nodal DOF are also coupled. Finally, Equation (22) is solved to obtain the iFEM reconstructed nodal displacements, U, of the structure.

## 3. Experimental Test Specimen

This section presents the geometric and material specifications of the panel specimen used for the experimental investigation. Additionally, details regarding the loading conditions, sensor configuration instrumented on the structure, and the inverse models used for the iFEM analysis are also discussed.

### 3.1. Composite Wing Panel

**Figure 3:**
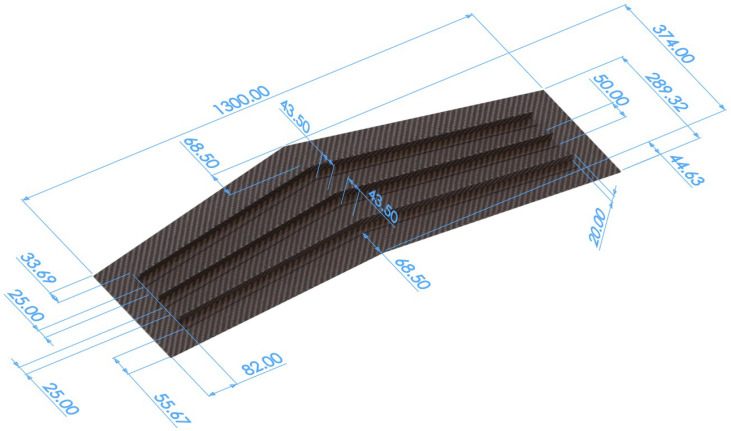
Geometry of the stiffened wing panel. All dimensions are expressed in mm.

The structure analyzed in this work is a composite stiffened wing panel. The panel is swept and is stiffened by three T-section stringers installed on one face. These stringers lie along the wing span and are not parallel to each other. The geometry of the panel is presented in Figure 3.

**Figure 4:**
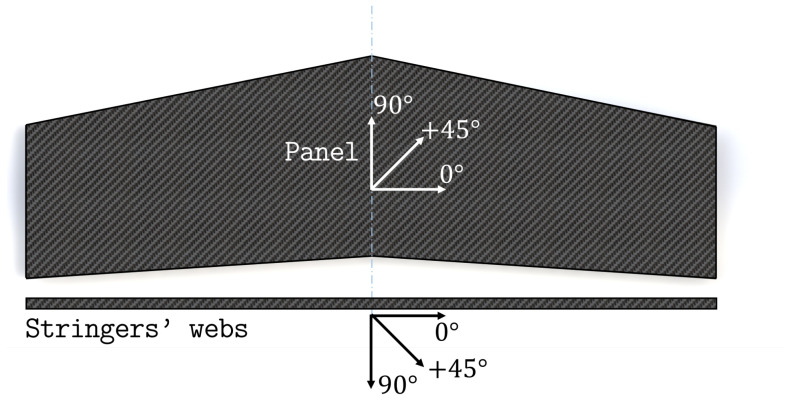
Reference directions for the panel’s lay-up orientations.

**Figure 5:**
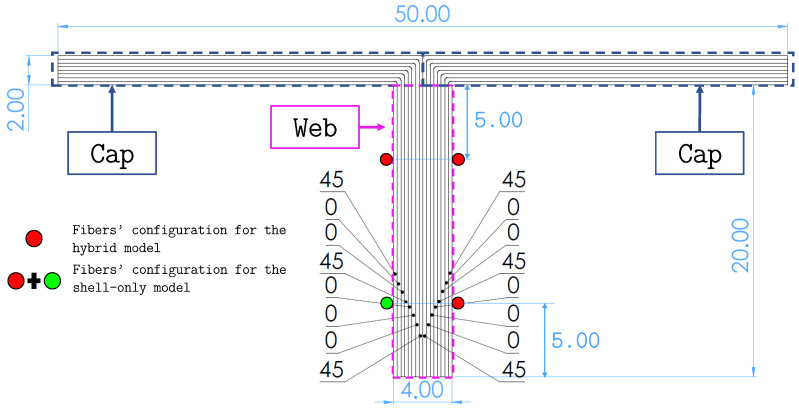
Transverse section of the stringers. The figure shows the stacking sequence of the stringer’s web and how it is folded to obtain the two caps. Moreover, the strain sensor configuration for the web is illustrated. All dimensions are expressed in mm.

The entire structure is made of a multilayered composite whose layers are made of a TWILL T-300 carbon-fiber fabric prepreg. The characteristics of the prepreg are reported in Table 1. The layup stacking sequence of the panel and of the web of the stringers is ${\left[45/0/0/45/0/0/0/45\right]}_{s}$, according to the reference directions shown in Figure 4. The T-section stringers are manufactured by bending the layers of the web at a 90${}^{\circ }$ angle to obtain the two caps, which are then glued to the panel (Figure 5). Therefore, the stacking sequence of each cap is derived by folding one half of the web’s stacking sequence.

### 3.2. Inverse Element Models

**Figure 6:**
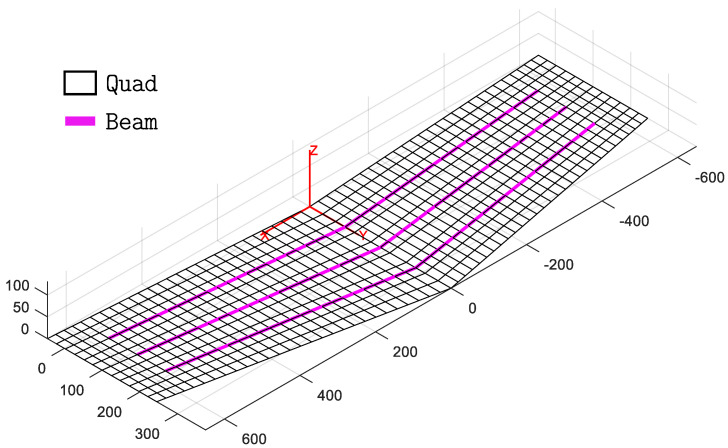
Hybrid inverse model of the panel.

The experimental investigation aims to validate the hybrid iFEM approach that combines both beam and shell elements within the same inverse model. Therefore, an inverse model of the wing panel, composed of the two-node 0th-order Timoshenko inverse beam element [52,54] and the four-node quadrilateral inverse shell element (iQS4) [23], is developed and shown in Figure 6.

The inverse quad elements are used to model the flat panel skin and the stringer caps, while the web of the stringers is modeled using inverse beam elements. The characteristics that have to be defined for these inverse elements are only related to their geometry, since the inverse model does not require any information about the material of the structure. Therefore, only the thickness of the quad elements and the cross-sectional properties of the beam elements need to be defined. The shell elements that simulate portions of the skin without underlying caps have a thickness of 4 mm as a result of the relative stacking sequence’s total thickness. The shell elements that model the regions of the panel where the skin and the caps of the stringers overlap have a thickness of 6 mm (4 mm for the skin and 2 mm for the cap). Finally, the beam elements that simulate the web of the stringers have a rectangular 4 × 20 mm${}^{2}$ cross-section (Figure 5). The whole model is constituted by 672 quad elements, 144 beam elements, and 741 nodes. Each node has 6 DOF, accounting for a total of 4446 DOF for the entire model.

**Figure 7:**
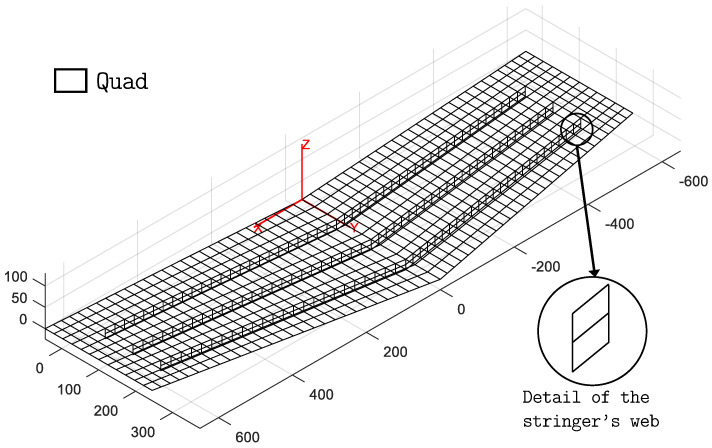
Shell-only inverse model of the panel.

A second inverse model is also developed to evaluate the relative advantages and disadvantages of the hybrid modeling scheme. It is a standard inverse model composed only of shell elements (Figure 7).

The panel skin and the stringer caps are simulated similarly to the hybrid model. Instead, the web of the stringers is modeled using two lines of 4 mm thick quad elements. This model is used as a benchmark to compare the accuracy and efficiency of the hybrid model. It is constituted by 960 quad elements and 1035 nodes, resulting in a total of 6210 DOF. The higher number of DOF with respect to the hybrid model indicates a more computationally demanding model to analyze.

A high-fidelity direct FE model of the structure is also derived from the second inverse model. It is obtained by splitting each element of the complete shell model into four quad elements, thus obtaining a refined mesh. The FE model is used to simulate the experimental tests and produce numerical data for the preliminary computations. The numerical strains and displacements obtained from the direct FE model are used to identify an optimal strain-sensor configuration for the iFEM analysis of the structure. This process is described in the following sections.

### 3.3. Test Configuration

**Figure 8:**
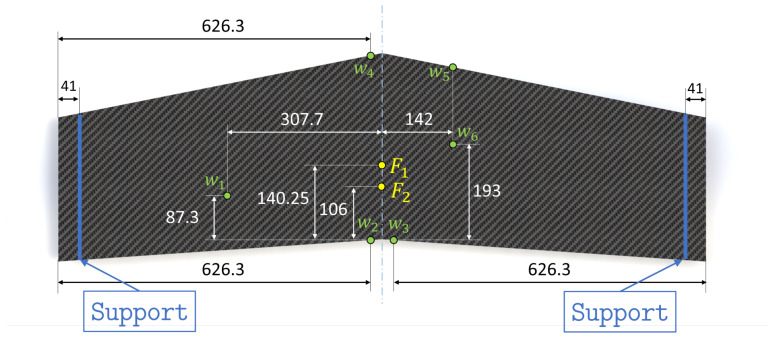
Testing configuration of the panel with the boundary conditions indicated. Moreover, the transverse loads $F_{1}$ and $F_{2}$, relative to the first and second loading conditions, are presented. The locations of the six transverse displacement transducers ($w_{1-6}$) are also shown. All dimensions are expressed in mm.

Due to its geometric complexity, the stiffened panel is prone to experience complex deformations and is a challenging test for shape sensing. Two loading conditions are considered for the experimental tests to simulate distinct panel behavior, as shown in Figure 8.

**Figure 9:**
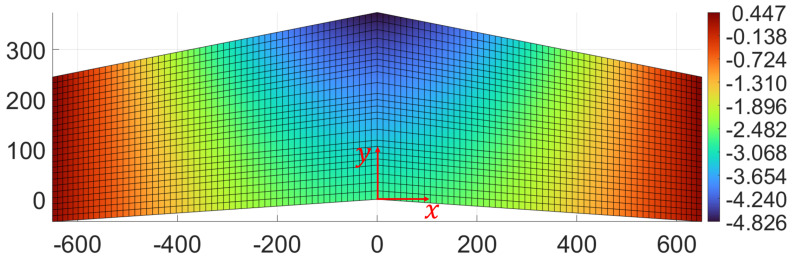
Torsional load case: contour plot of the transverse deformation (along *z*) for the first load case. All dimensions and displacements are expressed in mm.

Both load cases consider simply supported boundary conditions at the wing tips and a concentrated transverse force applied on the flat surface of the panel. The first load case ($F_{1}$, see Figure 8) is designed to generate a considerable amount of torsion in the structure. In fact, due to the swept nature of the wings, application of a concentrated transverse load of 200 N at the wing root, at a distance of 140.25 mm from the trailing edge, provokes significant torsional deformation. Figure 9 shows the plot of transverse deflection for the first load case obtained from a high-fidelity FE simulation.

**Figure 10:**
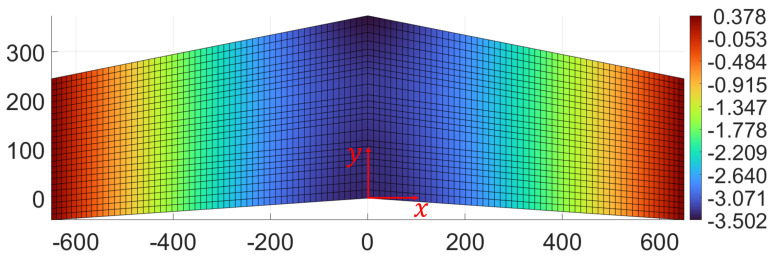
Primarily bending load case: contour plot of the transverse deformation (along *z*) for the second load case. All dimensions and displacements are expressed in mm.

The second load case ($F_{2}$, see Figure 8) is obtained by moving the concentrated force along the root section of the wing, at a distance of 106 mm from the trailing edge. In this case, bending deformation of the wing is predominant with respect to torsion (see Figure 10).

Therefore, the two load cases, although applied on the same structure, generate two considerably different problems for the shape-sensing procedure. The experimental setup also includes displacement transducers installed to measure the transverse displacement of the panel. Six LVDTs ($w_{1-6}$) are considered (see Figure 8). They are randomly distributed over the flat surface of the wing and are used as a reference to evaluate the accuracy of the iFEM reconstructed displacements.

### 3.4. Strain Sensors

The application of iFEM is based on the use of experimental strain measurements, with the sensor configuration strongly affecting the accuracy of the method. Before introducing the procedure used to select the optimal sensor configuration, it is important to understand how sensor selection and instrumentation are influenced by the different inverse elements and models. According to Equations (4) and (14), strain measurements from surface-mounted sensors can be used to compute the strain measures of the beam (see Equation (5)) and shell (see Equation (15)) element functionals. For this application, only axial stretching and bending curvatures of the beam elements are considered, while the transverse shear and torsional strain measures are assumed to be unknown. Therefore, for each sensorized beam element (see Equation (7)), the weighting coefficients $w_{k}^{0}$ (*k* = 1, 2, 3) are given a value of 1, while $w_{k}^{0}$ (*k* = 4, 5, 6) are set to a small value ($10^{-4}$). The strain measures are computed at two axial sections (at $l_{e}/4$ and $3l_{e}/4$) per element and require three surface strain measurements (oriented axially) at each section, resulting in a total of six strain measurements per element. The axial surface strains for the beam elements are measured by instrumenting the stringer’s web with three fiber-optic sensors (shown as red dots in Figure 5). These sensors run all along the length of the stringer and are capable of producing high-density strain measurements.

**Figure 11:**
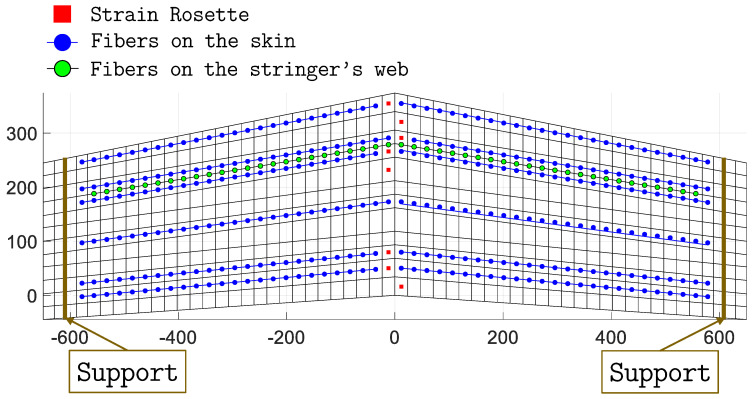
The optimal sensor configuration.

In contrast, the quadrilateral shell elements are instrumented using the scheme shown in Figure 2, where the strain sensors are placed in a back-to-back-configuration (i.e., every measurement point on the top surface of the panel has a corresponding one on the bottom). For each sensorized element, only strain sensors located at the centroid of the element are considered, according to the scheme adopted in Ref. [32]. Fiber-optic sensors are also used in this case, as shown by the blue dotted lines of the sensor configuration in Figure 11. High-density fiber-optic sensors running along the wing span are used to instrument multiple elements and measure strains only along the fiber direction. Therefore, if the local *x*-axis of each element is aligned with the corresponding fiber direction, the weighting coefficients of Equation (15) assume the value $w_{m}=w_{k}=\left\{1,10^{-5},10^{-5}\right\}$.

For the shell-only model, the webs of the stringers can be instrumented with fiber optics as well. In this case, considering the two lines of quad elements that model the web (see detail in Figure 7), measuring surface strains in a back-to-back configuration along the centroids of the two lines of elements requires an additional fourth fiber, shown as a green dot in Figure 5. As a consequence, the sensorization of the webs for the shell-only model is relatively more demanding in terms of strain sensors compared to the hybrid model. As strains are only measured along the fiber direction, even for the webs, if this direction coincides with the local *x*-axis of the element, the corresponding weighting coefficients assume the values $w_{m}=w_{k}=\left\{1,10^{-5},10^{-5}\right\}$.

Although the fibers allow a significant amount of surface strain to be measured, they are limited to measuring strains only in one direction (uni-axial), resulting in only one non-penalized term in the error functional. For this reason, some shell elements are instrumented with strain rosettes to measure tri-axial strains. Strain rosettes are considered for the two rows of elements across the chord length next to the root section of the panel, where the deformation is expected to be the highest. These rosettes allow the full characterization of the in-plane stretching and bending curvatures of the element. Therefore, for elements instrumented with rosettes, $w_{m}=w_{k}=\left\{1,1,1\right\}$.

Considering the instrumentation schemes mentioned above and the available sensors, an optimization procedure is used to identify an optimal sensor configuration for the iFEM analysis. Mainly fiber-optic sensors are considered for this application, as they can be used to efficiently sensorize both shell and beam elements. One 20 m fiber-optic sensor is considered. In addition, 16 strain rosettes, for measuring strains close to the wing root, are included in the optimization process.

The optimization is performed through the application of a genetic algorithm (GA) [63]. The objective of the optimization process is to simultaneously minimize two errors in the reconstructed displacements obtained from the iFEM analysis of the hybrid model. The first error considered is a local one. It is the percentage error in the reconstruction of the maximum transverse displacement of the panel, $\%Err_{w}^{max}$. The second error takes into account the global accuracy of the reconstructions over all the nodes of the inverse mesh. It is the percentage root mean squared error computed as follows [6,23]:

(23) $$\%Erms_{w}=100\times \sqrt{\frac{1}{N}\sum_{i=1}^{N}{\left(\frac{w_{i}-w_{i}^{ref}}{w_{max}^{ref}}\right)}^{2}}$$

where *N* is the number of nodes of the inverse mesh, $w_{i}$ are the reconstructed displacements, $w_{i}^{ref}$ are the reference displacements, and $w_{max}^{ref}$ is the maximum value of the reference displacements. The scaling of the error through the maximum value of the displacement, $w_{max}^{ref}$, allows the evaluation of the global accuracy, prioritizing the errors on larger and more significant displacements and reducing the influence of errors on the reconstruction of small displacements. For the optimization, only numerical data are used. The strain data and the reference displacements are computed through the FE analysis of the refined model of the structure. Only the torsional load case is considered. In fact, this work also aims to test the robustness of iFEM results obtained from a single sensor configuration with respect to the variability in load cases and, consequently, in the deformed shapes that need to be reconstructed. The GA used to perform the optimization considers 200 individuals and 2800 generations. For each generation, the one-point crossover, the two-point crossover, the mutation, and the permutation are applied with a probability of 1, 0.9, 0.001, and 0.001, respectively.

The optimized sensor configuration is shown in Figure 11. The error values for this configuration are $\%Err_{w}^{max}=0.17$ and $\%Erms_{w}=0.22$. The six lines of fiber and eight rosettes on the panel are instrumented in a back-to-back configuration for both inverse models. In contrast, sensorization of the stringer’s web requires two different sensor configurations: one with three fiber lines for the hybrid model, and one with four fiber lines for the shell-only model, according to the scheme discussed previously and shown in Figure 5.

## 4. Experimental Setup and Results

**Figure 12:**
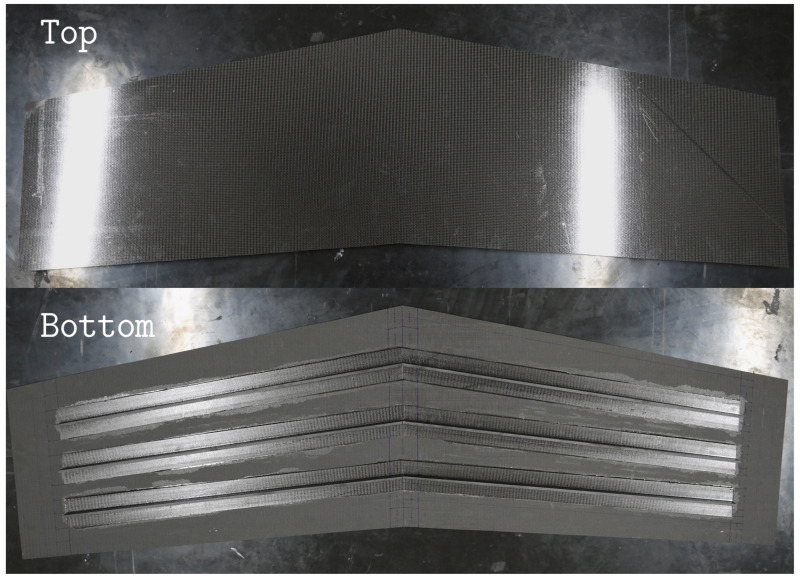
**Top** and **bottom** surfaces of the composite wing panel.

**Figure 13:**
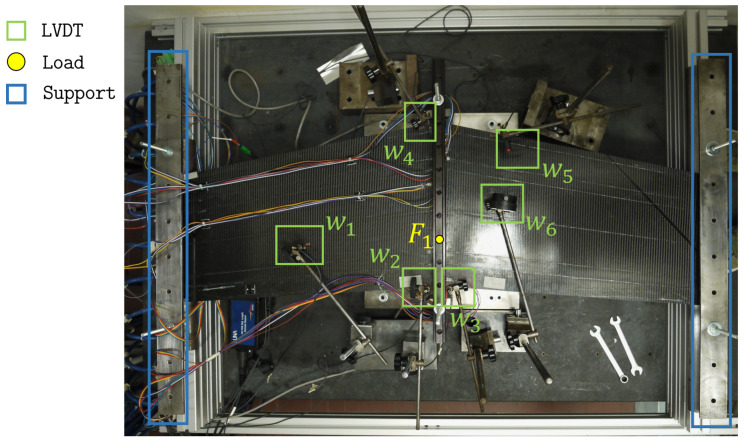
Experimental test setup showing the location of the LVDTs, loads, and supports.

The manufactured composite wing panel is shown in Figure 12, and the experimentally realized test setup, as described in Section 3.3, is shown in Figure 13.

**Figure 14:**
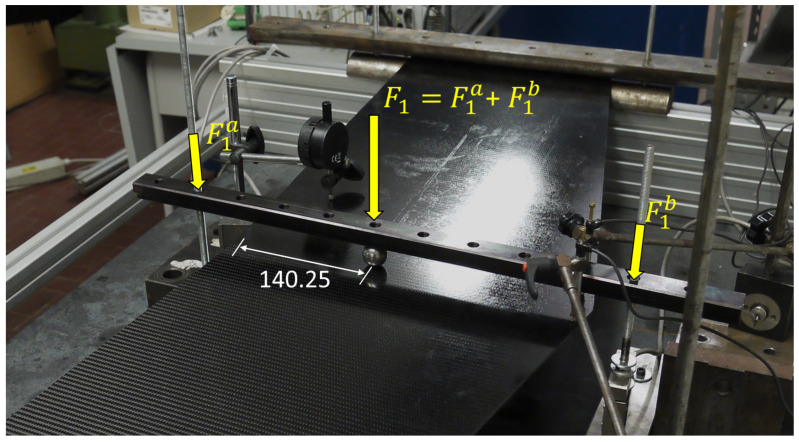
The load application system for the first load case. The sphere that transmits the load can be moved along the bar to obtain the second load case.

The simply supported condition is realized by placing the two sections close to the extreme edges of the panel between two half-cylinder iron bars. Since the curved surfaces of the half cylinders are in contact with the panel, the transverse displacements are constrained, whereas the out-of-plane rotations are not. The concentrated forces for the two load cases are applied on the structure through an iron sphere pressed against the flat surface of the panel (see Figure 14). An iron bar, connected between two threaded bars, is placed on top of the sphere and can be moved down by tightening two bolts on the supporting bars. The bar transfers the load to the sphere, which transmits it to the panel. The load is measured by two load cells connected to the threaded bars. Moreover, the sphere can be moved along the bar, and consequently along the root section of the panel, thus allowing the realization of both loading configurations.

The stiffened panel is instrumented with two 10-meter-long optic fiber strain sensors based on Rayleigh scattering and optical frequency domain reflectometry (OFDR). This approach considers the fiber itself as a sensor by detecting changes in the characteristics of the light scattered along the fiber length, caused by the local variation of strain [64]. These high-density sensors allow strains along the fiber direction to be measured with a density of 1 every 1.3 mm. By realizing multiple loops on the structure with the fibers, the configuration shown in Figure 11 is experimentally realized. To complete the sensor configuration, 16 tri-axial strain rosettes (8 locations in a back-to-back configuration) are instrumented at the prescribed locations close to the wing root. The experimental setup is completed with 6 LVDTs that measure the transverse displacements at the locations mentioned in Figure 8. These sensors are located on the flat surface of the panel (TOP) and are used to experimentally assess the accuracy of the iFEM reconstructions.

Multiple tests based on the two load cases were performed, and strain and displacement data were collected. Consistency of measurements between successive tests indicated their reliability. Therefore, considering the small variability between the results of each test, the results of only one test per load case are presented here. Moreover, the comparison between results obtained with the hybrid model and the shell-only model are performed considering the same set of data. In Table 2, the experimental values of the measured transverse deflections and the ones reconstructed using either the hybrid or the shell-only inverse model are reported for the two different load cases. Additionally, the percentage error in iFEM results for each displacement ($\%Err_{w_{i}}$), together with the mean of the absolute value of the errors (μ(|%Err|)), are also reported.

Analyzing the errors for the torsional load case highlights the accuracy of the iFEM analysis. In fact, the errors never exceed 8% for both inverse models. The maximum error achieved using the hybrid model is 5.2%, whereas the one achieved by the shell-only model is 8%. Moreover, the error relative to the maximum measured displacement, $w_{4}$, shows a similar difference. In this case, the error of the hybrid model is only $\%Err_{w_{4}}=-2.0\%$, versus the higher $\%Err_{w_{4}}=-6.5\%$ of the shell-only model. Overall, the mean error, μ(|%Err|), also shows a slight advantage in favor of the hybrid model, with an error 1% lower than the shell-only model. In conclusion, the level of accuracy achieved by the hybrid model for the torsional load case is better than that of the shell-only model.

The primarily bending load case is fundamental in this application for two reasons. The first objective is to assess the adaptability of the iFEM in general to the reconstruction of different deformed shapes using the same sensor configuration. In fact, the same sensor configuration optimized for the first load case is adopted for the second one. The second objective is to consider another application to specifically evaluate the accuracy of the introduced hybrid formulation. For the first objective, the results reported in Table 2 demonstrate impressive robustness. In fact, although the configuration is optimized for the torsional load case, the errors obtained by the two models for the bending case are comparable with the ones obtained for the primary load case, in terms of the individual and mean errors.

Comparing the two models for the bending load case leads to similar conclusions as those of the previous one. The maximum error of the hybrid model (5%) is lower than the shell-only model (7.0%) in this case as well. The error in maximum deflection reconstruction, $w_{4}$, is also in favor of the hybrid model, with a value of $\%Err_{w_{4}}=-1.9\%$ compared to $\%Err_{w_{4}}=-7.0\%$ for the shell-only model. The overall accuracy, measured by the mean error, is also comparable between the two models, with a difference of only 1.2% in favor of the hybrid model. These evaluations also prove the slightly higher accuracy of the hybrid model. In conclusion, for a comprehensive comparison of the two models, it is important to note that the novel hybrid model is able to achieve a higher accuracy using a more computationally efficient model with a lower number of DOF and relatively lower number of strain sensors than the standard shell-only modeling approach.

## 5. Conclusions

This work presented and experimentally validated a novel hybrid formulation of the inverse finite element method. This formulation improves existing modeling capabilities of this shape-sensing method by introducing the possibility of creating hybrid models, i.e., models that discretize the structure using beam and shell inverse elements. Such a scheme is particularly adaptable for modeling thin-walled stiffened panels where the panel can be modeled using shell elements and the stringers using beam elements within the same inverse model. Compared to standard shell-only models, hybrid models are more computationally efficient and require a relatively lower number of strain sensors to produce shape-sensing results of similar accuracy.

This hybrid iFEM formulation is evaluated experimentally for the shape sensing of a composite stiffened wing panel. The accuracy of the novel formulation is assessed by comparing the results with those obtained from a standard shell-only model. Two different loading conditions are considered: one that induces combined torsion and bending deformation, and a second that is primarily bending. The panel is instrumented with strain rosettes and fiber-optic sensors whose optimal configuration is obtained through an optimization scheme considering numerical results only from the torsional load case. The experimental results prove that the hybrid model is able to achieve consistently accurate displacement reconstructions for both load cases. Comparison with the shell-only model successfully demonstrated that the hybrid model achieves a comparable, or slightly greater, accuracy while using a more computationally efficient model and relatively lower number of strain sensors. Moreover, the use of a single sensor configuration across the two load cases demonstrated the adaptability of iFEM to variability in the analyzed deformation field.

Although the results presented for the hybrid iFEM are promising, future adoption of this shape-sensing methodology relies on its ability to adapt to sparse sensor configurations. In fact, the sensor requirements for the shape-sensing techniques represent a strong limitation for the monitoring of existing structures, either those instrumented with few sensors or those which present difficulties in the installation of new ones. Future research in this field should focus on techniques that further reduce the number of strain sensors required. Additionally, efforts should be directed at demonstrating the applicability of iFEM to real-world scenarios by monitoring increasingly complex structures with reduced availability of strain sensors.

## Figures and Tables

**Table 1:** TWILL T-300 nominal properties.

$E_{11}$ [GPa]	$E_{22}$ [GPa]	$\nu_{12}$	$G_{12}=G_{23}=G_{13}$ [GPa]	Thickness [mm]
59.7	59.7	0.09	3.8	0.25

**Table 2:** Shape sensing results: experimentally measured and reconstructed transverse displacements are reported for the two load cases. In parentheses, the percentage errors with respect to the experimental values are reported. Moreover, the mean of the absolute value of the percentage error is also reported (μ(|%Err|)).

	Torsional Load Case			Primarily Bending Load Case		
	Experimental	Hybrid iFEM	Shell-Only iFEM	Experimental	Hybrid iFEM	Shell-Only iFEM
*F* [N]	200			200		
$w_{1}$ [mm]	2.42	2.52	2.50	2.44	2.56	2.55
($\%Err_{w_{1}}$)		(+4.3%)	(+3.5%)		(+5.0%)	(+4.2%)
$w_{2}$ [mm]	2.98	3.14	3.14	3.72	3.89	3.89
($\%Err_{w_{2}}$)		(+5.2%)	(+5.4%)		(+4.5%)	(+4.5%)
$w_{3}$ [mm]	2.98	3.13	3.14	3.72	3.89	3.90
($\%Err_{w_{3}}$)		(+5.3%)	(+5.4%)		(+4.7%)	(+4.8%)
$w_{4}$ [mm]	5.18	5.07	4.84	3.77	3.70	3.51
($\%Err_{w_{4}}$)		(−2.0%)	(−6.5%)		(−1.9%)	(−7.0%)
$w_{5}$ [mm]	4.72	4.54	4.35	3.42	3.35	3.19
($\%Err_{w_{5}}$)		(−3.9%)	(−8.0%)		(−2.2%)	(−6.8%)
$w_{6}$ [mm]	3.75	3.90	3.82	3.29	3.42	3.36
($\%Err_{w_{6}}$)		(+3.9%)	(+1.8%)		(+3.9%)	(+1.9%)
μ(|%Err|)		4.1%	5.1%		3.7%	4.9%

## Data Availability

The data presented in this study are available on request from the corresponding author.
